# *catena*-Poly[{μ_3_-4,4′,6,6′-tetra­bromo-2,2′-[butane-1,4-diylbis(nitrilo­methan­ylyl­idene)]diphenolato}{μ_2_-4,4′,6,6′-tetra­bromo-2,2′-[butane-1,4-diylbis(nitrilo­methanylyl­idene)]dipheno­lato}dicopper(II)]

**DOI:** 10.1107/S1600536812029285

**Published:** 2012-06-30

**Authors:** Hadi Kargar, Reza Kia, Amir Adabi Ardakani, Muhammad Nawaz Tahir

**Affiliations:** a Department of Chemistry, Payame Noor University, PO BOX 19395-3697 Tehran, I. R. of IRAN; b Department of Chemistry, Science and Research Branch, Islamic Azad University, Tehran, Iran; c Structural Dynamics of (Bio)Chemical Systems, Max Planck Institute for Biophysical Chemistry, Am Fassberg 11, 37077 Göttingen, Germany; d Department of Physics, University of Sargodha, Punjab, Pakistan

## Abstract

The asymmetric unit of the title coordination polymer consists of a dinuclear neutral complex mol­ecule of formula [Cu_2_(C_18_H_14_Br_4_N_2_O_2_)_2_]_*n*_. One of the Cu^II^ ions is coordinated in a distorted square-planar geometry, whereas the other is coordinated in a distorted square-pyramidal geometry, the long apical Cu—O bond [2.885 (4) Å] of the square-pyramidal coordination being provided by a symmetry-related O atom creating a one-dimensional polymer along [010]. π–π stacking inter­actions [centroid–centroid distance = 3.783 (4) Å] and short inter­chain Br⋯Br inter­actions [3.6142 (12)–3.6797 (12) Å] are observed.

## Related literature

For standard bond lengths, see: Allen *et al.* (1987 ▶). For van der Waals radii, see: Bondi (1964 ▶). For background to coordination polymers, see: Kido & Okamoto (2002 ▶); Li *et al.* (2006 ▶). For background to bis-bidentate Schiff base complexes, see: Hannon *et al.* (1999 ▶); Lavalette *et al.* (2003 ▶). For the synthesis and structural variations of Schiff base complexes, see: Granovski *et al.* (1993 ▶); Elmali *et al.* (2000 ▶). For related structures, see: Kargar & Kia (2011**a* ▶,b* ▶).
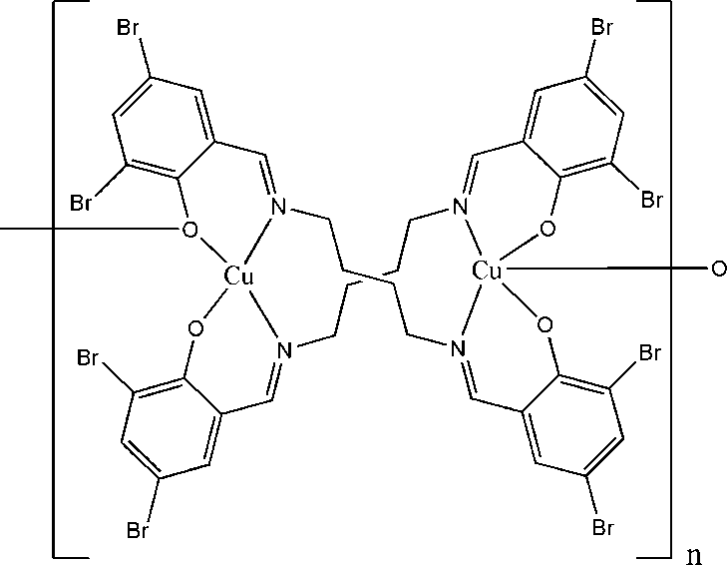


## Experimental

### 

#### Crystal data

[Cu_2_(C_18_H_14_Br_4_N_2_O_2_)_2_]
*M* *_r_* = 1345.98
Orthorhombic, 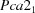

*a* = 27.4100 (11) Å
*b* = 7.9055 (4) Å
*c* = 18.8291 (7) Å
*V* = 4080.1 (3) Å^3^
*Z* = 4
Mo *K*α radiation
μ = 8.92 mm^−1^
*T* = 291 K
0.36 × 0.18 × 0.16 mm

#### Data collection

Bruker SMART APEXII CCD area-detector diffractometer
Absorption correction: multi-scan (*SADABS*; Bruker, 2001 ▶) *T*_min_ = 0.142, *T*_max_ = 0.329
33878 measured reflections
8854 independent reflections
5887 reflections with *I* > 2σ(*I*)
*R*_int_ = 0.071

#### Refinement

*R*[*F* ^2^ > 2σ(*F* ^2^)] = 0.042
*wR*(*F* ^2^) = 0.068
*S* = 1.00
8854 reflections
487 parameters
1 restraint
H-atom parameters constrained
Δρ_max_ = 0.55 e Å^−3^
Δρ_min_ = −0.45 e Å^−3^
Absolute structure: Flack (1983 ▶), 4178 Friedel pairs
Flack parameter: 0.069 (8)

### 

Data collection: *APEX2* (Bruker, 2007 ▶); cell refinement: *SAINT* (Bruker, 2007 ▶); data reduction: *SAINT*; program(s) used to solve structure: *SHELXS97* (Sheldrick, 2008 ▶); program(s) used to refine structure: *SHELXL97* (Sheldrick, 2008 ▶); molecular graphics: *SHELXTL* (Sheldrick, 2008 ▶); software used to prepare material for publication: *SHELXTL* and *PLATON* (Spek, 2009 ▶).

## Supplementary Material

Crystal structure: contains datablock(s) global, I. DOI: 10.1107/S1600536812029285/rz2776sup1.cif

Structure factors: contains datablock(s) I. DOI: 10.1107/S1600536812029285/rz2776Isup2.hkl

Additional supplementary materials:  crystallographic information; 3D view; checkCIF report

## Figures and Tables

**Table 1:** Hydrogen-bond geometry (Å, °)

*D*—H⋯*A*	*D*—H	H⋯*A*	*D*⋯*A*	*D*—H⋯*A*
C10—H10*B*⋯O3	0.97	2.50	3.099 (7)	120
C27—H27*B*⋯O2	0.97	2.54	3.129 (7)	119

**Fig. 1.**
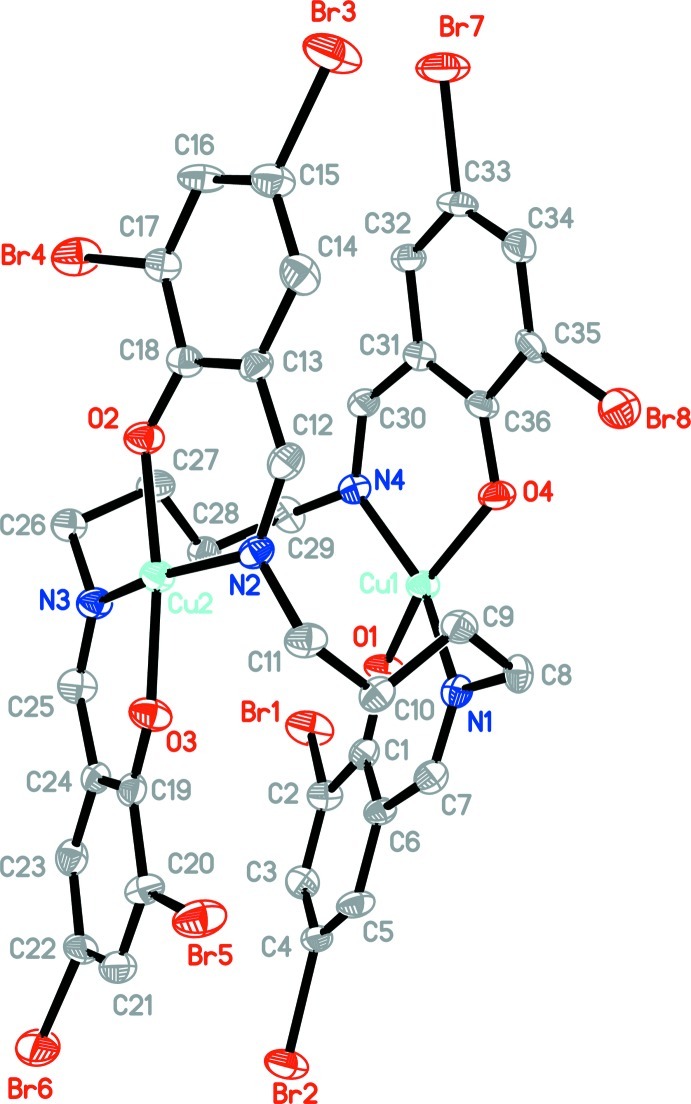
The molecular structure of the title complex, showing 40% probability displacement ellipsoids. H atoms have been omitted for clarity.

**Fig. 2.**
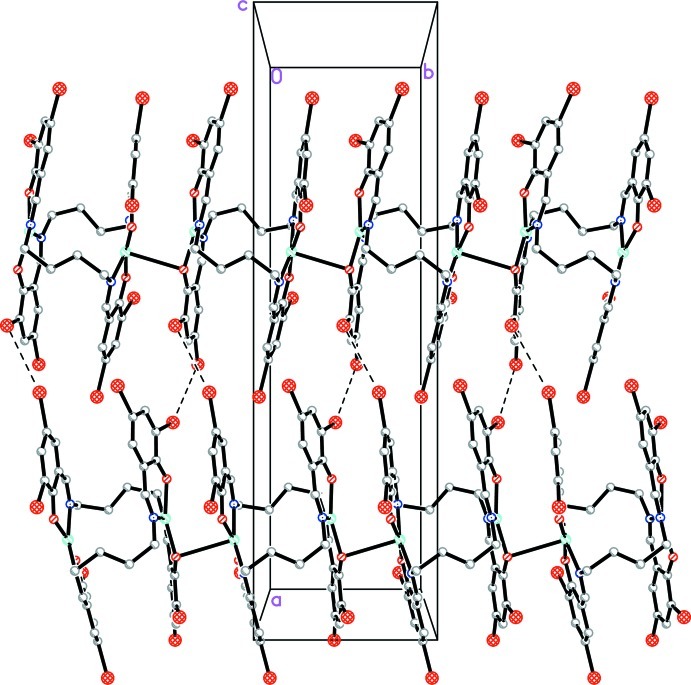
Partial crystal packing, viewed down the c axis, of the title complex showing the one-dimensional coordination chain propagating along the b axis. Br···Br interactions are shown as dashed lines. H atoms have been omitted for clarity.

## supplementary crystallographic information

### Comment

The design and construction of metal-organic coordination polymers (MOCPs) have attracted considerable attention, not only for their novel topologies but also for their potential in the area of magnetic applications and functional materials (Kido & Okamoto, 2002; Li *et al.*, 2006). One of the key strategies in the construction of metal-organic coordination polymers is to select suitable bi- or multi-dentate bridging ligands. Among these, bis-bidentate *NN*- or *NO*-donor Schiff base ligands with aliphatic and aromatic spacers (Hannon *et al.*, 1999; Lavalette *et al.*, 2003) have attracted much attention because of the flexibility in their coordination modes and the resulting intermolecular interactions. The long chain aliphatic spacers or rigid aromatic spacers with large bite angles in these ligands favour the bis-bidentate coordination mode and allow the ligands to accomodate metal centers in one unit of the ligand. On the other hand, Schiff bases are one of the most prevalent ligands in coordination chemistry and their complexes are some of the most important stereochemical models in transition metal-organic chemistry, with their ease of preparation and structural variations (Granovski *et al.*, 1993; Elmali *et al.*, 2000).

The molecular structure of the title complex (Fig. 1) consists of dinuclear units in which the Schiff base ligands are twisted around copper(II) metal centres in a bis-bidentate coordination mode. The bond lengths (Allen *et al.,* 1987) and angles are within normal ranges and are comparable to those reported for related structures (Kargar & Kia, 2011**a*,b*). Both metal atoms show a tetrahedrally distorted square-planar coordination geometry provided by two nitrogen and two oxygen atoms of the Schiff-base ligands. Two C—H···O hydrogen bonds (Table 1) stabilize the conformation of the complex molecule. A fifth coordination site is provided for atom Cu2 by the O4 oxygen atom of a neighbouring complex forming one-dimensional coordination polymeric chains along the *b* axis (Fig. 2). The length of the Cu2—O4^i^ bond [2.885 (4)Å; symmetry code: (i) x, 1 + y, z] is shorter than the sum of the van der Waals radii of these atoms [Cu, 1.43Å and O, 1.52 Å; Bondi, 1964]. The chains are further stabilized π-π stacking interactions [*Cg*1···*Cg*2^ii^ = 3.736 (2) Å; symmetry code (ii) x, -1 + y, z; *Cg*1 and *Cg*2 are the centroids of the C1–C6 and C19–C24 rings, respectively]. In the crystal structure, short interchain Br···Br contacts are observed [Br(1)···Br(7)^iii^ = 3.6797 (12) Å; Br(2)···Br(4)^iv^ = 3.6142 (12) Å; Br(4)···Br(6)^v^ = 3.6142 (12) Å; Br(6)···Br(8)^vi^ = 3.6401 (12) Å; symmetry codes: (iii) -1/2 + x, 1 - y, z; (iv) 1/2 - x, y, 1/2 + z; (v) 1/2 - x, y, -1/2 + z; (vi) -1/2 + x, 2 - y, z]. A Br(8)···C(12)^ii^ [3.447 (6) Å] interaction is also present in the crystal structure which is shorter than sum of the van der Waals radii of Br [3.70Å] and C [1.70 Å] atoms (Bondi, 1964).

### Experimental

The title complex was synthesized by the reaction of an methanolic solution (50 ml) of bis(3,5-bromosalicylaldeyde)-1,4-butanediimine (2 mmol) and CuCl_2_.4H_2_O (2 mmol). After stirring at reflux conditions for 2 h, the solution was filtered and the resulting dark-brown powder was crystallized from DMF, giving single crystals suitable for X-ray diffraction.

### Refinement

All H atoms were positioned geometrically and constrained to refine with their parent atoms using a riding-model approximation, with C—H = 0.93-0.97 Å and *U*_iso_(H) = 1.2 *U*_eq_(C).

### Crystal data

**Table:**

[Cu_2_(C_18_H_14_Br_4_N_2_O_2_)_2_]	*F*(000) = 2564
*M**_r_* = 1345.98	*D*_x_ = 2.191 Mg m^−^^3^
Orthorhombic, *P**c**a*2_1_	Mo *K*α radiation, λ = 0.71073 Å
Hall symbol: P 2c -2ac	Cell parameters from 2567 reflections
*a* = 27.4100 (11) Å	θ = 2.5–27.7°
*b* = 7.9055 (4) Å	µ = 8.92 mm^−^^1^
*c* = 18.8291 (7) Å	*T* = 291 K
*V* = 4080.1 (3) Å^3^	Needle, dark-brown
*Z* = 4	0.36 × 0.18 × 0.16 mm

### Data collection

**Table:**

Bruker SMART APEXII CCD area-detector diffractometer	8854 independent reflections
Radiation source: fine-focus sealed tube	5887 reflections with *I* > 2σ(*I*)
Graphite monochromator	*R*_int_ = 0.071
φ and ω scans	θ_max_ = 27.2°, θ_min_ = 1.5°
Absorption correction: multi-scan (*SADABS*; Bruker, 2001)	*h* = −35→35
*T*_min_ = 0.142, *T*_max_ = 0.329	*k* = −10→7
33878 measured reflections	*l* = −24→24

### Refinement

**Table:**

Refinement on *F*^2^	Secondary atom site location: difference Fourier map
Least-squares matrix: full	Hydrogen site location: inferred from neighbouring sites
*R*[*F*^2^ > 2σ(*F*^2^)] = 0.042	H-atom parameters constrained
*wR*(*F*^2^) = 0.068	*w* = 1/[σ^2^(*F*_o_^2^) + (0.0125*P*)^2^] where *P* = (*F*_o_^2^ + 2*F*_c_^2^)/3
*S* = 1.00	(Δ/σ)_max_ = 0.001
8854 reflections	Δρ_max_ = 0.55 e Å^−^^3^
487 parameters	Δρ_min_ = −0.45 e Å^−^^3^
1 restraint	Absolute structure: Flack (1983), 4178 Friedel pairs
Primary atom site location: structure-invariant direct methods	Flack parameter: 0.069 (8)

### Special details

**Table:**

Geometry. All esds (except the esd in the dihedral angle between two l.s. planes) are estimated using the full covariance matrix. The cell esds are taken into account individually in the estimation of esds in distances, angles and torsion angles; correlations between esds in cell parameters are only used when they are defined by crystal symmetry. An approximate (isotropic) treatment of cell esds is used for estimating esds involving l.s. planes.
Refinement. Refinement of F^2^ against ALL reflections. The weighted R-factor wR and goodness of fit S are based on F^2^, conventional R-factors R are based on F, with F set to zero for negative F^2^. The threshold expression of F^2^ > 2sigma(F^2^) is used only for calculating R-factors(gt) etc. and is not relevant to the choice of reflections for refinement. R-factors based on F^2^ are statistically about twice as large as those based on F, and R- factors based on ALL data will be even larger.

### Fractional atomic coordinates and isotropic or equivalent isotropic displacement parameters (Å^2^)

**Table:**

	*x*	*y*	*z*	*U*_iso_*/*U*_eq_	
Cu1	0.33709 (3)	0.59065 (10)	0.49527 (4)	0.03293 (19)	
Cu2	0.37343 (2)	1.17409 (10)	0.50858 (4)	0.03289 (19)	
Br1	0.17168 (3)	0.56192 (11)	0.40823 (4)	0.0593 (2)	
Br2	0.10462 (3)	0.83048 (10)	0.66708 (4)	0.0541 (2)	
Br3	0.62640 (3)	0.96666 (13)	0.39602 (5)	0.0732 (3)	
Br4	0.44790 (3)	1.16280 (11)	0.27446 (4)	0.0561 (2)	
Br5	0.30469 (3)	1.27351 (11)	0.74365 (4)	0.0578 (2)	
Br6	0.11632 (3)	1.30310 (11)	0.62177 (5)	0.0632 (2)	
Br7	0.56876 (3)	0.57140 (14)	0.29593 (4)	0.0761 (3)	
Br8	0.50186 (2)	0.48746 (10)	0.57844 (4)	0.05028 (19)	
O1	0.26874 (14)	0.5837 (6)	0.4829 (2)	0.0381 (11)	
O2	0.41492 (15)	1.1542 (6)	0.4276 (2)	0.0405 (12)	
O3	0.33343 (14)	1.2315 (5)	0.5877 (2)	0.0404 (11)	
O4	0.40368 (14)	0.5235 (5)	0.5055 (2)	0.0402 (11)	
N1	0.32999 (17)	0.6362 (6)	0.5991 (2)	0.0308 (13)	
N2	0.42674 (17)	1.1050 (6)	0.5741 (2)	0.0310 (12)	
N3	0.31486 (19)	1.1816 (7)	0.4438 (3)	0.0368 (14)	
N4	0.34345 (17)	0.6214 (6)	0.3909 (2)	0.0306 (12)	
C1	0.2344 (2)	0.6384 (8)	0.5247 (3)	0.0339 (16)	
C2	0.1857 (2)	0.6452 (8)	0.5005 (3)	0.0377 (16)	
C3	0.1483 (2)	0.7013 (8)	0.5410 (3)	0.0390 (17)	
H3	0.1167	0.7031	0.5230	0.047*	
C4	0.1575 (2)	0.7563 (8)	0.6100 (3)	0.0356 (16)	
C5	0.2036 (2)	0.7512 (8)	0.6359 (3)	0.0398 (17)	
H5	0.2093	0.7868	0.6822	0.048*	
C6	0.24245 (19)	0.6945 (8)	0.5954 (3)	0.0300 (15)	
C7	0.2895 (2)	0.6784 (8)	0.6279 (3)	0.0354 (16)	
H7	0.2908	0.7015	0.6763	0.042*	
C8	0.3727 (2)	0.6193 (8)	0.6465 (3)	0.0338 (16)	
H8A	0.3904	0.5168	0.6346	0.041*	
H8B	0.3616	0.6094	0.6952	0.041*	
C9	0.4068 (2)	0.7694 (8)	0.6403 (3)	0.0349 (17)	
H9A	0.4358	0.7475	0.6684	0.042*	
H9B	0.4169	0.7810	0.5912	0.042*	
C10	0.3844 (2)	0.9332 (8)	0.6645 (3)	0.0367 (16)	
H10A	0.3777	0.9263	0.7149	0.044*	
H10B	0.3536	0.9495	0.6400	0.044*	
C11	0.4172 (2)	1.0860 (9)	0.6505 (3)	0.0391 (17)	
H11A	0.4016	1.1874	0.6684	0.047*	
H11B	0.4479	1.0717	0.6755	0.047*	
C12	0.4703 (2)	1.0696 (8)	0.5534 (3)	0.0372 (17)	
H12	0.4922	1.0414	0.5892	0.045*	
C13	0.4896 (2)	1.0676 (8)	0.4826 (3)	0.0334 (16)	
C14	0.5388 (2)	1.0243 (9)	0.4740 (3)	0.0440 (18)	
H14	0.5574	0.9950	0.5135	0.053*	
C15	0.5599 (2)	1.0248 (9)	0.4078 (4)	0.0479 (19)	
C16	0.5327 (2)	1.0691 (9)	0.3491 (3)	0.0452 (19)	
H16	0.5470	1.0714	0.3043	0.054*	
C17	0.4846 (2)	1.1095 (8)	0.3570 (3)	0.0354 (17)	
C18	0.4607 (2)	1.1130 (8)	0.4237 (3)	0.0334 (16)	
C19	0.2864 (2)	1.2337 (8)	0.5952 (3)	0.0315 (15)	
C20	0.2645 (2)	1.2575 (8)	0.6626 (3)	0.0388 (17)	
C21	0.2150 (2)	1.2745 (8)	0.6710 (4)	0.0444 (18)	
H21	0.2021	1.2931	0.7160	0.053*	
C22	0.1841 (2)	1.2639 (9)	0.6129 (4)	0.0408 (18)	
C23	0.2034 (2)	1.2353 (8)	0.5477 (4)	0.0389 (17)	
H23	0.1826	1.2261	0.5088	0.047*	
C24	0.2540 (2)	1.2192 (8)	0.5373 (3)	0.0331 (16)	
C25	0.2710 (2)	1.2002 (9)	0.4655 (3)	0.0407 (18)	
H25	0.2471	1.2017	0.4304	0.049*	
C26	0.3207 (2)	1.1622 (8)	0.3660 (3)	0.0369 (17)	
H26A	0.3478	1.2310	0.3499	0.044*	
H26B	0.2914	1.2019	0.3423	0.044*	
C27	0.3299 (2)	0.9758 (9)	0.3458 (3)	0.0408 (18)	
H27A	0.3369	0.9704	0.2954	0.049*	
H27B	0.3588	0.9373	0.3708	0.049*	
C28	0.2886 (2)	0.8547 (9)	0.3619 (4)	0.0417 (18)	
H28A	0.2796	0.8666	0.4114	0.050*	
H28B	0.2605	0.8859	0.3334	0.050*	
C29	0.3009 (2)	0.6721 (8)	0.3476 (3)	0.0370 (17)	
H29	0.2841	0.6019	0.3163	0.044*	
C30	0.3837 (2)	0.6062 (8)	0.3574 (3)	0.0363 (17)	
H30	0.3821	0.6166	0.3083	0.044*	
C31	0.4315 (2)	0.5745 (8)	0.3870 (3)	0.0362 (16)	
C32	0.4705 (2)	0.5838 (8)	0.3391 (3)	0.0404 (17)	
H32	0.4647	0.6030	0.2911	0.048*	
C33	0.5173 (2)	0.5642 (10)	0.3635 (4)	0.049 (2)	
C34	0.5266 (2)	0.5366 (8)	0.4343 (4)	0.0432 (18)	
H34	0.5586	0.5253	0.4501	0.052*	
C35	0.4886 (2)	0.5256 (8)	0.4817 (3)	0.0365 (17)	
C36	0.4388 (2)	0.5406 (8)	0.4602 (3)	0.0361 (17)	

### Atomic displacement parameters (Å^2^)

**Table:**

	*U*^11^	*U*^22^	*U*^33^	*U*^12^	*U*^13^	*U*^23^
Cu1	0.0269 (4)	0.0410 (5)	0.0308 (4)	0.0002 (4)	0.0004 (3)	−0.0006 (4)
Cu2	0.0310 (4)	0.0391 (5)	0.0286 (4)	0.0032 (4)	−0.0009 (3)	−0.0020 (4)
Br1	0.0431 (4)	0.0917 (7)	0.0429 (4)	0.0022 (4)	−0.0096 (3)	−0.0238 (4)
Br2	0.0415 (4)	0.0759 (6)	0.0448 (4)	0.0152 (4)	0.0078 (3)	−0.0095 (4)
Br3	0.0422 (5)	0.1092 (8)	0.0683 (5)	0.0223 (5)	0.0063 (4)	−0.0097 (5)
Br4	0.0487 (4)	0.0878 (7)	0.0318 (4)	−0.0031 (4)	−0.0042 (3)	−0.0035 (4)
Br5	0.0506 (5)	0.0907 (7)	0.0321 (4)	−0.0025 (4)	0.0023 (3)	−0.0020 (4)
Br6	0.0347 (4)	0.0777 (7)	0.0770 (5)	0.0032 (4)	0.0163 (4)	0.0150 (5)
Br7	0.0415 (5)	0.1278 (9)	0.0589 (5)	0.0105 (5)	0.0207 (4)	0.0162 (5)
Br8	0.0361 (4)	0.0671 (6)	0.0476 (4)	0.0007 (4)	−0.0064 (3)	0.0057 (4)
O1	0.025 (2)	0.057 (3)	0.033 (2)	−0.001 (2)	0.0020 (18)	−0.012 (2)
O2	0.036 (3)	0.058 (4)	0.028 (2)	0.005 (2)	0.0009 (19)	0.001 (2)
O3	0.030 (3)	0.056 (3)	0.035 (2)	0.005 (2)	−0.002 (2)	−0.008 (2)
O4	0.027 (2)	0.058 (3)	0.035 (3)	0.002 (2)	0.005 (2)	0.007 (2)
N1	0.029 (3)	0.027 (4)	0.036 (3)	−0.008 (2)	−0.001 (2)	0.001 (2)
N2	0.033 (3)	0.032 (3)	0.028 (3)	−0.008 (3)	0.002 (2)	−0.002 (2)
N3	0.037 (3)	0.046 (4)	0.027 (3)	0.003 (3)	0.001 (2)	−0.001 (3)
N4	0.026 (3)	0.036 (4)	0.030 (3)	−0.006 (2)	−0.003 (2)	−0.002 (2)
C1	0.035 (4)	0.031 (5)	0.036 (4)	−0.007 (3)	0.001 (3)	0.004 (3)
C2	0.033 (4)	0.047 (5)	0.033 (4)	−0.003 (3)	−0.001 (3)	−0.005 (3)
C3	0.029 (4)	0.045 (5)	0.044 (4)	−0.002 (3)	−0.004 (3)	0.000 (3)
C4	0.034 (4)	0.047 (5)	0.027 (4)	0.008 (3)	0.005 (3)	0.004 (3)
C5	0.040 (4)	0.058 (5)	0.022 (3)	0.001 (3)	0.005 (3)	−0.001 (3)
C6	0.023 (3)	0.038 (4)	0.029 (3)	−0.005 (3)	0.002 (3)	0.001 (3)
C7	0.037 (4)	0.041 (5)	0.028 (3)	−0.003 (3)	−0.004 (3)	0.000 (3)
C8	0.032 (4)	0.032 (5)	0.038 (4)	−0.001 (3)	−0.010 (3)	0.004 (3)
C9	0.025 (3)	0.040 (5)	0.040 (4)	−0.003 (3)	0.000 (3)	0.000 (3)
C10	0.039 (4)	0.041 (5)	0.030 (3)	−0.003 (3)	−0.001 (3)	0.003 (3)
C11	0.040 (4)	0.054 (5)	0.024 (3)	−0.001 (4)	−0.001 (3)	−0.006 (3)
C12	0.042 (4)	0.040 (5)	0.030 (4)	−0.004 (3)	−0.003 (3)	0.002 (3)
C13	0.033 (4)	0.032 (5)	0.035 (4)	−0.005 (3)	0.004 (3)	0.003 (3)
C14	0.039 (4)	0.051 (5)	0.042 (4)	0.006 (4)	−0.010 (3)	−0.003 (3)
C15	0.042 (4)	0.063 (6)	0.039 (4)	0.007 (4)	0.008 (4)	−0.005 (4)
C16	0.035 (4)	0.068 (6)	0.033 (4)	0.000 (4)	0.011 (3)	−0.014 (4)
C17	0.034 (4)	0.041 (5)	0.032 (3)	−0.001 (3)	−0.001 (3)	−0.006 (3)
C18	0.032 (4)	0.033 (5)	0.035 (4)	−0.003 (3)	0.003 (3)	−0.008 (3)
C19	0.036 (4)	0.026 (4)	0.032 (4)	−0.002 (3)	0.001 (3)	0.002 (3)
C20	0.041 (4)	0.045 (5)	0.031 (3)	−0.006 (3)	0.008 (3)	−0.002 (3)
C21	0.052 (5)	0.037 (5)	0.044 (4)	−0.002 (3)	0.017 (4)	−0.003 (4)
C22	0.027 (4)	0.039 (5)	0.056 (5)	0.003 (3)	0.014 (3)	0.004 (4)
C23	0.034 (4)	0.039 (5)	0.044 (4)	0.000 (3)	−0.005 (3)	0.006 (3)
C24	0.040 (4)	0.032 (5)	0.027 (3)	0.004 (3)	0.001 (3)	0.004 (3)
C25	0.033 (4)	0.056 (5)	0.033 (4)	0.001 (4)	−0.008 (3)	−0.004 (3)
C26	0.033 (4)	0.052 (5)	0.026 (3)	0.004 (3)	−0.006 (3)	0.001 (3)
C27	0.041 (4)	0.049 (5)	0.032 (4)	−0.003 (4)	0.001 (3)	−0.007 (3)
C28	0.033 (4)	0.049 (5)	0.043 (4)	0.004 (3)	0.005 (3)	0.009 (4)
C29	0.037 (4)	0.039 (5)	0.035 (4)	0.001 (3)	−0.015 (3)	−0.011 (3)
C30	0.039 (4)	0.044 (5)	0.026 (3)	0.005 (3)	0.005 (3)	0.004 (3)
C31	0.032 (4)	0.038 (5)	0.038 (4)	0.003 (3)	0.006 (3)	0.002 (3)
C32	0.031 (4)	0.055 (5)	0.035 (4)	0.007 (3)	0.007 (3)	0.009 (4)
C33	0.030 (4)	0.065 (6)	0.050 (4)	0.009 (4)	0.019 (3)	0.006 (4)
C34	0.032 (4)	0.039 (5)	0.059 (5)	0.002 (3)	0.004 (3)	0.008 (4)
C35	0.030 (4)	0.031 (5)	0.049 (4)	0.011 (3)	0.002 (3)	−0.001 (3)
C36	0.026 (4)	0.035 (5)	0.047 (4)	0.003 (3)	0.009 (3)	0.000 (3)

### Geometric parameters (Å, º)

**Table:**

Cu1—O1	1.889 (4)	C10—H10A	0.9700
Cu1—O4	1.911 (4)	C10—H10B	0.9700
Cu1—N4	1.989 (5)	C11—H11A	0.9700
Cu1—N1	1.997 (5)	C11—H11B	0.9700
Cu2—O3	1.905 (4)	C12—C13	1.435 (8)
Cu2—O2	1.909 (4)	C12—H12	0.9300
Cu2—N2	1.989 (5)	C13—C14	1.402 (8)
Cu2—N3	2.016 (5)	C13—C18	1.409 (8)
Br1—C2	1.897 (6)	C14—C15	1.373 (8)
Br2—C4	1.899 (6)	C14—H14	0.9300
Br3—C15	1.892 (6)	C15—C16	1.380 (9)
Br4—C17	1.899 (6)	C16—C17	1.363 (8)
Br5—C20	1.887 (6)	C16—H16	0.9300
Br6—C22	1.890 (6)	C17—C18	1.417 (8)
Br7—C33	1.901 (6)	C19—C24	1.411 (8)
Br8—C35	1.882 (6)	C19—C20	1.416 (8)
O1—C1	1.301 (7)	C20—C21	1.374 (8)
O2—C18	1.299 (7)	C21—C22	1.387 (9)
O3—C19	1.297 (7)	C21—H21	0.9300
O4—C36	1.293 (7)	C22—C23	1.356 (8)
N1—C7	1.279 (7)	C23—C24	1.406 (8)
N1—C8	1.479 (6)	C23—H23	0.9300
N2—C12	1.286 (7)	C24—C25	1.437 (8)
N2—C11	1.469 (7)	C25—H25	0.9300
N3—C25	1.279 (7)	C26—C27	1.543 (9)
N3—C26	1.482 (7)	C26—H26A	0.9700
N4—C30	1.277 (7)	C26—H26B	0.9700
N4—C29	1.479 (7)	C27—C28	1.513 (8)
C1—C2	1.411 (8)	C27—H27A	0.9700
C1—C6	1.421 (8)	C27—H27B	0.9700
C2—C3	1.352 (8)	C28—C29	1.506 (9)
C3—C4	1.393 (8)	C28—H28A	0.9700
C3—H3	0.9300	C28—H28B	0.9700
C4—C5	1.355 (8)	C29—H29	0.9300
C5—C6	1.383 (7)	C30—C31	1.444 (8)
C5—H5	0.9300	C30—H30	0.9300
C6—C7	1.434 (7)	C31—C32	1.401 (8)
C7—H7	0.9300	C31—C36	1.418 (9)
C8—C9	1.514 (8)	C32—C33	1.372 (8)
C8—H8A	0.9700	C32—H32	0.9300
C8—H8B	0.9700	C33—C34	1.374 (9)
C9—C10	1.503 (8)	C34—C35	1.374 (8)
C9—H9A	0.9700	C34—H34	0.9300
C9—H9B	0.9700	C35—C36	1.429 (8)
C10—C11	1.528 (8)		
			
O1—Cu1—O4	162.17 (19)	C15—C14—C13	120.6 (6)
O1—Cu1—N4	88.22 (18)	C15—C14—H14	119.7
O4—Cu1—N4	92.86 (18)	C13—C14—H14	119.7
O1—Cu1—N1	91.66 (18)	C14—C15—C16	120.0 (6)
O4—Cu1—N1	92.55 (18)	C14—C15—Br3	120.8 (5)
N4—Cu1—N1	162.58 (19)	C16—C15—Br3	119.3 (5)
O3—Cu2—O2	170.95 (19)	C17—C16—C15	119.7 (6)
O3—Cu2—N2	90.16 (19)	C17—C16—H16	120.2
O2—Cu2—N2	92.04 (18)	C15—C16—H16	120.2
O3—Cu2—N3	90.44 (19)	C16—C17—C18	123.3 (6)
O2—Cu2—N3	89.63 (19)	C16—C17—Br4	118.4 (5)
N2—Cu2—N3	165.5 (2)	C18—C17—Br4	118.3 (5)
C1—O1—Cu1	129.4 (4)	O2—C18—C13	124.2 (5)
C18—O2—Cu2	129.9 (4)	O2—C18—C17	120.2 (5)
C19—O3—Cu2	131.3 (4)	C13—C18—C17	115.6 (6)
C36—O4—Cu1	128.0 (4)	O3—C19—C24	122.8 (5)
C7—N1—C8	117.0 (5)	O3—C19—C20	121.3 (5)
C7—N1—Cu1	123.2 (4)	C24—C19—C20	115.9 (5)
C8—N1—Cu1	119.9 (4)	C21—C20—C19	122.4 (6)
C12—N2—C11	116.1 (5)	C21—C20—Br5	118.5 (5)
C12—N2—Cu2	123.6 (4)	C19—C20—Br5	119.1 (4)
C11—N2—Cu2	120.3 (4)	C20—C21—C22	120.4 (6)
C25—N3—C26	115.3 (5)	C20—C21—H21	119.8
C25—N3—Cu2	124.0 (4)	C22—C21—H21	119.8
C26—N3—Cu2	120.6 (4)	C23—C22—C21	119.1 (6)
C30—N4—C29	115.9 (5)	C23—C22—Br6	119.4 (5)
C30—N4—Cu1	123.5 (4)	C21—C22—Br6	121.4 (5)
C29—N4—Cu1	120.6 (4)	C22—C23—C24	121.8 (6)
O1—C1—C2	120.2 (5)	C22—C23—H23	119.1
O1—C1—C6	123.8 (5)	C24—C23—H23	119.1
C2—C1—C6	116.0 (5)	C23—C24—C19	120.4 (6)
C3—C2—C1	123.2 (6)	C23—C24—C25	117.4 (6)
C3—C2—Br1	118.5 (5)	C19—C24—C25	122.0 (6)
C1—C2—Br1	118.2 (5)	N3—C25—C24	128.1 (6)
C2—C3—C4	119.4 (6)	N3—C25—H25	115.9
C2—C3—H3	120.3	C24—C25—H25	115.9
C4—C3—H3	120.3	N3—C26—C27	111.1 (5)
C5—C4—C3	119.8 (6)	N3—C26—H26A	109.4
C5—C4—Br2	121.2 (4)	C27—C26—H26A	109.4
C3—C4—Br2	119.0 (5)	N3—C26—H26B	109.4
C4—C5—C6	121.9 (5)	C27—C26—H26B	109.4
C4—C5—H5	119.1	H26A—C26—H26B	108.0
C6—C5—H5	119.1	C28—C27—C26	115.6 (5)
C5—C6—C1	119.8 (5)	C28—C27—H27A	108.4
C5—C6—C7	119.0 (5)	C26—C27—H27A	108.4
C1—C6—C7	120.9 (5)	C28—C27—H27B	108.4
N1—C7—C6	128.5 (5)	C26—C27—H27B	108.4
N1—C7—H7	115.8	H27A—C27—H27B	107.4
C6—C7—H7	115.8	C29—C28—C27	113.8 (5)
N1—C8—C9	111.8 (5)	C29—C28—H28A	108.8
N1—C8—H8A	109.3	C27—C28—H28A	108.8
C9—C8—H8A	109.3	C29—C28—H28B	108.8
N1—C8—H8B	109.3	C27—C28—H28B	108.8
C9—C8—H8B	109.3	H28A—C28—H28B	107.7
H8A—C8—H8B	107.9	N4—C29—C28	109.7 (5)
C10—C9—C8	113.6 (5)	N4—C29—H29	125.1
C10—C9—H9A	108.8	C28—C29—H29	125.1
C8—C9—H9A	108.8	N4—C30—C31	127.5 (6)
C10—C9—H9B	108.8	N4—C30—H30	116.2
C8—C9—H9B	108.8	C31—C30—H30	116.2
H9A—C9—H9B	107.7	C32—C31—C36	121.9 (6)
C9—C10—C11	112.9 (5)	C32—C31—C30	115.7 (6)
C9—C10—H10A	109.0	C36—C31—C30	122.4 (5)
C11—C10—H10A	109.0	C33—C32—C31	119.4 (6)
C9—C10—H10B	109.0	C33—C32—H32	120.3
C11—C10—H10B	109.0	C31—C32—H32	120.3
H10A—C10—H10B	107.8	C32—C33—C34	121.1 (6)
N2—C11—C10	110.7 (5)	C32—C33—Br7	117.8 (5)
N2—C11—H11A	109.5	C34—C33—Br7	121.1 (5)
C10—C11—H11A	109.5	C33—C34—C35	120.0 (6)
N2—C11—H11B	109.5	C33—C34—H34	120.0
C10—C11—H11B	109.5	C35—C34—H34	120.0
H11A—C11—H11B	108.1	C34—C35—C36	122.3 (6)
N2—C12—C13	128.8 (6)	C34—C35—Br8	119.6 (5)
N2—C12—H12	115.6	C36—C35—Br8	118.1 (5)
C13—C12—H12	115.6	O4—C36—C31	123.7 (6)
C14—C13—C18	120.8 (6)	O4—C36—C35	121.0 (6)
C14—C13—C12	117.7 (6)	C31—C36—C35	115.2 (5)
C18—C13—C12	121.4 (6)		
			
O4—Cu1—O1—C1	120.3 (6)	C12—C13—C14—C15	−178.1 (6)
N4—Cu1—O1—C1	−145.9 (5)	C13—C14—C15—C16	0.3 (11)
N1—Cu1—O1—C1	16.7 (5)	C13—C14—C15—Br3	179.9 (5)
N2—Cu2—O2—C18	−1.2 (5)	C14—C15—C16—C17	−1.1 (11)
N3—Cu2—O2—C18	−166.7 (5)	Br3—C15—C16—C17	179.3 (5)
N2—Cu2—O3—C19	−152.3 (5)	C15—C16—C17—C18	1.7 (11)
N3—Cu2—O3—C19	13.2 (5)	C15—C16—C17—Br4	−178.1 (5)
O1—Cu1—O4—C36	108.0 (7)	Cu2—O2—C18—C13	2.1 (9)
N4—Cu1—O4—C36	14.9 (5)	Cu2—O2—C18—C17	−178.7 (4)
N1—Cu1—O4—C36	−148.6 (5)	C14—C13—C18—O2	179.9 (6)
O1—Cu1—N1—C7	−12.7 (5)	C12—C13—C18—O2	−2.2 (10)
O4—Cu1—N1—C7	−175.4 (5)	C14—C13—C18—C17	0.6 (9)
N4—Cu1—N1—C7	76.6 (8)	C12—C13—C18—C17	178.5 (6)
O1—Cu1—N1—C8	166.4 (4)	C16—C17—C18—O2	179.3 (6)
O4—Cu1—N1—C8	3.8 (4)	Br4—C17—C18—O2	−0.9 (8)
N4—Cu1—N1—C8	−104.2 (7)	C16—C17—C18—C13	−1.4 (10)
O3—Cu2—N2—C12	−170.4 (5)	Br4—C17—C18—C13	178.3 (5)
O2—Cu2—N2—C12	0.8 (5)	Cu2—O3—C19—C24	−11.7 (9)
N3—Cu2—N2—C12	97.2 (9)	Cu2—O3—C19—C20	170.7 (5)
O3—Cu2—N2—C11	11.8 (4)	O3—C19—C20—C21	174.4 (6)
O2—Cu2—N2—C11	−177.0 (4)	C24—C19—C20—C21	−3.4 (9)
N3—Cu2—N2—C11	−80.6 (9)	O3—C19—C20—Br5	−3.6 (8)
O3—Cu2—N3—C25	−7.0 (6)	C24—C19—C20—Br5	178.7 (5)
O2—Cu2—N3—C25	−177.9 (5)	C19—C20—C21—C22	1.9 (10)
N2—Cu2—N3—C25	85.3 (10)	Br5—C20—C21—C22	179.9 (5)
O3—Cu2—N3—C26	174.2 (5)	C20—C21—C22—C23	0.5 (10)
O2—Cu2—N3—C26	3.2 (5)	C20—C21—C22—Br6	−175.3 (5)
N2—Cu2—N3—C26	−93.5 (9)	C21—C22—C23—C24	−1.2 (10)
O1—Cu1—N4—C30	−166.8 (5)	Br6—C22—C23—C24	174.7 (5)
O4—Cu1—N4—C30	−4.6 (5)	C22—C23—C24—C19	−0.4 (10)
N1—Cu1—N4—C30	103.3 (8)	C22—C23—C24—C25	−176.0 (6)
O1—Cu1—N4—C29	16.1 (4)	O3—C19—C24—C23	−175.1 (6)
O4—Cu1—N4—C29	178.2 (4)	C20—C19—C24—C23	2.6 (9)
N1—Cu1—N4—C29	−73.8 (8)	O3—C19—C24—C25	0.2 (10)
Cu1—O1—C1—C2	169.9 (4)	C20—C19—C24—C25	177.9 (6)
Cu1—O1—C1—C6	−9.9 (9)	C26—N3—C25—C24	178.9 (6)
O1—C1—C2—C3	−179.7 (6)	Cu2—N3—C25—C24	0.0 (10)
C6—C1—C2—C3	0.1 (10)	C23—C24—C25—N3	−179.1 (7)
O1—C1—C2—Br1	3.2 (8)	C19—C24—C25—N3	5.5 (11)
C6—C1—C2—Br1	−177.0 (4)	C25—N3—C26—C27	−103.0 (6)
C1—C2—C3—C4	0.3 (10)	Cu2—N3—C26—C27	76.0 (6)
Br1—C2—C3—C4	177.3 (5)	N3—C26—C27—C28	64.1 (7)
C2—C3—C4—C5	−0.7 (10)	C26—C27—C28—C29	−174.7 (5)
C2—C3—C4—Br2	−178.7 (5)	C30—N4—C29—C28	−105.3 (6)
C3—C4—C5—C6	0.8 (10)	Cu1—N4—C29—C28	72.0 (6)
Br2—C4—C5—C6	178.8 (5)	C27—C28—C29—N4	58.9 (7)
C4—C5—C6—C1	−0.5 (10)	C29—N4—C30—C31	173.5 (6)
C4—C5—C6—C7	−174.6 (6)	Cu1—N4—C30—C31	−3.7 (9)
O1—C1—C6—C5	179.8 (6)	N4—C30—C31—C32	−172.6 (6)
C2—C1—C6—C5	0.0 (9)	N4—C30—C31—C36	5.8 (11)
O1—C1—C6—C7	−6.2 (9)	C36—C31—C32—C33	−1.8 (10)
C2—C1—C6—C7	174.0 (6)	C30—C31—C32—C33	176.6 (6)
C8—N1—C7—C6	−176.3 (6)	C31—C32—C33—C34	−0.4 (11)
Cu1—N1—C7—C6	2.9 (9)	C31—C32—C33—Br7	178.2 (5)
C5—C6—C7—N1	−176.5 (6)	C32—C33—C34—C35	1.0 (11)
C1—C6—C7—N1	9.5 (10)	Br7—C33—C34—C35	−177.6 (5)
C7—N1—C8—C9	−103.6 (6)	C33—C34—C35—C36	0.5 (10)
Cu1—N1—C8—C9	77.2 (6)	C33—C34—C35—Br8	179.9 (6)
N1—C8—C9—C10	64.5 (7)	Cu1—O4—C36—C31	−16.8 (9)
C8—C9—C10—C11	−173.8 (5)	Cu1—O4—C36—C35	163.3 (5)
C12—N2—C11—C10	−108.8 (6)	C32—C31—C36—O4	−176.7 (6)
Cu2—N2—C11—C10	69.2 (6)	C30—C31—C36—O4	5.0 (10)
C9—C10—C11—N2	61.0 (7)	C32—C31—C36—C35	3.1 (9)
C11—N2—C12—C13	176.4 (6)	C30—C31—C36—C35	−175.2 (6)
Cu2—N2—C12—C13	−1.4 (10)	C34—C35—C36—O4	177.4 (6)
N2—C12—C13—C14	180.0 (6)	Br8—C35—C36—O4	−2.0 (8)
N2—C12—C13—C18	2.0 (11)	C34—C35—C36—C31	−2.5 (10)
C18—C13—C14—C15	−0.1 (10)	Br8—C35—C36—C31	178.1 (5)

### Hydrogen-bond geometry (Å, º)

**Table:**

*D*—H···*A*	*D*—H	H···*A*	*D*···*A*	*D*—H···*A*
C10—H10*B*···O3	0.97	2.50	3.099 (7)	120
C27—H27*B*···O2	0.97	2.54	3.129 (7)	119
